# Dysregulation of Trace Elements in Pediatric Cholestasis: From Pathophysiology to Nutritional Approaches

**DOI:** 10.3390/ijms27062710

**Published:** 2026-03-16

**Authors:** Sorina Adam, Alina Grama, Alexandra Mititelu, Gabriel Benţa, Tudor Lucian Pop

**Affiliations:** 1 “Iuliu Hațieganu” University of Medicine and Pharmacy, 400012 Cluj-Napoca, Romania; adam.rogozan.sorina@elearn.umfcluj.ro; 2 2nd Pediatric Discipline, Department of Mother and Child, Faculty of Medicine, “Iuliu Hațieganu” University of Medicine and Pharmacy, 400012 Cluj-Napoca, Romania; 3 2nd Pediatric Clinic, Emergency Clinical Hospital for Children, 400124 Cluj-Napoca, Romania

**Keywords:** trace elements, cholestasis, metabolism, function, children

## Abstract

Cholestasis in children is characterized by impaired bile flow that disrupts hepatic metabolism, nutrient homeostasis, and effects trace element balance. This narrative review summarizes current evidence on the metabolism, biological functions, and clinical implications of key trace elements—zinc, selenium, copper, and manganese—in pediatric cholestatic liver disease. The liver regulates trace element absorption, intracellular trafficking, storage, and biliary excretion; cholestasis alters these processes, leading to deficiencies or toxic accumulation. Zinc and selenium deficiencies are common and contribute to impaired growth, immune dysfunction, oxidative stress, and delayed hepatic regeneration. Conversely, reduced biliary excretion promotes copper and manganese accumulation, potentially exacerbating liver injury and causing manganese-related neurotoxicity. Recent advances in understanding metal-specific hepatic transporters and trafficking pathways have provided mechanistic insight into these alterations. Management strategies emphasize individualized supplementation, monitoring during enteral and parenteral nutrition, and prevention of deficiency and toxicity. Precision-based nutritional approaches may improve outcomes in pediatric cholestatic liver disease.

## 1. Introduction

Trace elements are essential for key metabolic processes and the maintenance of normal physiological functions. These include chromium (Cr), copper (Cu), iodine (I), iron (Fe), manganese (Mn), molybdenum (Mo), selenium (Se), and zinc (Zn). These vital trace elements are involved in various biochemical reactions that support physiological functions and help regulate homeostasis [1]. These processes, including enzyme activity, cellular signaling, and immune function, play critical roles in maintaining homeostasis and supporting growth and development in children. The liver regulates the absorption, distribution, storage, and excretion of substances. This regulation is particularly crucial during childhood, a period of rapid growth and development requiring optimal micronutrient availability.

Trace elements are primarily absorbed in the duodenum and jejunum and transported via the portal circulation, where they bind to plasma proteins. The liver plays an important role in synthesizing specific carrier proteins that transport and distribute these trace elements to target tissues and organs. Additionally, the liver contributes to the excretion of certain elements, such as copper and magnesium, through bile production, underscoring its dual role in trace element regulation and detoxification [2].

Cholestasis, a common pediatric liver disorder characterized by impaired bile flow, disrupts trace element balance. The condition alters the enterohepatic circulation of bile acids and associated nutrients, leading to malabsorption of fat-soluble vitamins and trace elements. Zinc and selenium deficiencies, in particular, have been linked to immune dysfunction, poor growth, and delayed wound healing, while copper and iron imbalances may contribute to oxidative stress and tissue damage. In cholestatic liver diseases, such as biliary atresia and progressive familial intrahepatic cholestasis, trace element imbalances are further exacerbated by systemic inflammation and protein-energy malnutrition. Moreover, oxidative stress is exacerbated by the depletion of trace elements such as selenium and zinc, thereby amplifying hepatocellular injury.

Despite the significant clinical implications, trace element homeostasis in pediatric cholestasis remains underexplored. Understanding alterations in trace element metabolism can provide valuable insights into disease mechanisms and inform therapeutic strategies to mitigate complications and improve outcomes.

Although trace elements are not routinely assessed in clinical practice, their dysregulation may contribute to disease-related complications, impaired growth, and nutritional deficiencies, supporting the clinical relevance of addressing trace element metabolism in this population.

This review examines the roles of trace elements (Zn, Se, Cu, and Mn) in pediatric cholestasis, focusing on their absorption, metabolism, and clinical implications. By highlighting current knowledge and identifying research gaps, this article seeks to advance understanding of trace element homeostasis in children with cholestatic liver disease. We also aim to present evidence-based strategies for managing trace element imbalances in pediatric cholestasis, with a focus on optimizing outcomes.

This narrative review is based on a comprehensive literature search conducted in PubMed, Scopus, and Web of Science, focusing on studies published in English. Relevant articles were identified using combinations of keywords related to pediatric cholestasis, chronic liver disease, and trace elements (zinc, selenium, copper, and manganese). Original research articles, clinical studies, and relevant reviews were considered, with particular attention to pediatric populations; when pediatric data were limited, evidence from adult studies was included and clearly identified.

## 2. Zinc

Zinc is an essential trace element required for normal growth and development. It is the second-most abundant trace element after iron and has important antioxidant, anti-inflammatory, and anti-apoptotic effects [3].

Zinc is found in protein-rich foods such as meat (beef, turkey, chicken, and pork), crustaceans (crabs and oysters), dairy products, tree nuts (cashews and almonds), legumes (beans), and whole grains. It is primarily absorbed in the jejunum via the specific transporter Zip4 and subsequently excreted into bile with high efficiency [4,5]. Its absorption can be significantly influenced by the concurrent intake of other minerals, such as iron and copper, as well as by dietary components like phytate and fiber, which can reduce zinc’s bioavailability. Approximately 85% of the body’s zinc is stored in skeletal muscle and bone, with only about 0.1% circulating in plasma. Adjustments in intestinal absorption and excretion predominantly regulate zinc homeostasis. In plasma, zinc is primarily transported bound to albumin. Urinary excretion of zinc is typically less than 10% of the amount excreted in feces, except in cases of elevated muscle protein catabolism [6].

### 2.1. Functions of Zinc

Zinc is a trace element with crucial biological functions, including cellular, metabolic, and immune processes related to cell division, growth, and development [7]. The role of zinc in the body can be grouped into three general functional classes: structural, catalytic, and regulatory [6].

Zinc serves as a structural component in proteins. Zinc finger proteins are implicated in the transcription of DNA into RNA. The key roles of zinc in protein and nucleic acid synthesis explain its importance to growth and wound healing [8].

As a catalytic cofactor, zinc is present in more than 300 metalloenzymes that play essential roles in almost all metabolic pathways [8]. It is present in six main enzyme classes: oxidoreductases, transferases, hydrolases, lyases, isomerases, and ligases [8].

Zinc plays a critical role in regulating immune functions at various stages of immune responses. It contributes to multiple components of the antioxidant defense system, serving as a structural element in cytoplasmic superoxide dismutase, stabilizing cell membranes, and inducing the synthesis of metallothionein, which neutralizes reactive oxygen species [8]. Additionally, it contributes to cellular growth and differentiation, gene expression, and metabolic processes, and acts as a signaling mediator within endocrine, paracrine, and autocrine systems. For instance, it modulates insulin dynamics by decreasing insulin secretion and inhibiting hepatic insulin clearance.

### 2.2. Metabolism of Zinc in the Liver

The liver plays a central role in regulating zinc homeostasis and maintaining total body zinc balance. Zinc metabolism in the liver is intricately linked to hepatic function and is affected by any disruption in the liver parenchyma [7]. Under normal conditions, zinc is tightly regulated within hepatocytes, where it contributes to various biochemical and physiological processes [4,9]. The liver not only stores and distributes zinc but also regulates its release response to physiological demands or pathophysiological states [10,11].

Zinc homeostasis in the liver involves rapid exchange of zinc ions within hepatocytes, with turnover occurring within less than 2 days [4]. This dynamic process allows the liver to respond quickly to changes in zinc status and metabolic requirements. In conditions of increased metabolic activity or stress, such as inflammation, zinc metabolism is often transiently dysregulated, leading to reduced plasma zinc levels [7].

Hepatic zinc homeostasis is controlled through a complex network of zinc transporters and metallothioneins (MTs) that tightly regulate zinc uptake, distribution, storage, and efflux [12,13]. Transporters belonging to the ZnT (*SLC30*) family mediate zinc export into intracellular vesicles or the extracellular space, whereas the ZIP (*SLC39*) family promotes zinc influx into the cytoplasm from extracellular sources or intracellular compartments [12,13].

In hepatocytes, excess zinc is stored in MTs, small cysteine-rich proteins that bind zinc ions [14]. MTs act as intracellular buffers, ensuring adequate zinc availability for enzymatic and structural functions while preventing cytotoxic accumulation. During systemic inflammation, ZIP14 upregulation enhances hepatic zinc uptake, contributing to transient hypozincemia observed during acute-phase responses [13].

Zinc’s role in the liver extends to gene regulation, where it modulates the expression of multiple genes, including those for metallothionein, retinol-binding protein, cholecystokinin, and endothelin [7,15]. These genes are responsive to shifts in zinc concentration, with changes in their mRNA expression levels correlating with zinc availability. MTs are acute-phase proteins crucial for zinc absorption, intracellular storage, and distribution [12]. The increased intake of zinc triggers an increase in metallothionein synthesis [15].

**Figure 1:**
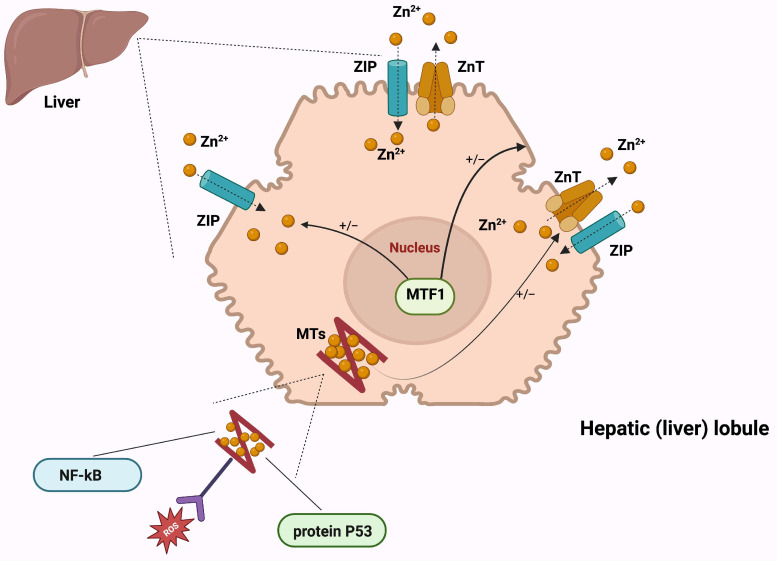
Cellular mechanisms of zinc sensing, transport, and signaling in hepatocytes: MTF1 (metal-responsive transcription factor 1) acts as a zinc-dependent nuclear sensor that activates or represses zinc-responsive genes, such as ZIP10, according to intracellular zinc availability. ZIP (*SLC39*) family transporters mediate zinc uptake into the cytoplasm, while ZnT (*SLC30*) transporters export zinc out of the cell or into specific intracellular compartments to maintain homeostasis. MTs serve as dynamic intracellular zinc buffers and donors, enabling zinc transfer to regulatory proteins, including p53, a tumor-suppressor transcription factor essential for genome stability and DNA repair, and NF-κB, a major transcriptional regulator of inflammation and immune responses [12,13,14]. Created in BioRender. Adam, S. (2026) https://BioRender.com/tj4oj1c.

Moreover, zinc influences several metabolic pathways in the liver, such as gluconeogenesis, and participates in the synthesis of acute-phase proteins and volatile substrates, such as nitric oxide [16]. A schematic representation of the mechanisms involved in zinc metabolism is presented in Figure 1.

### 2.3. Clinical Implications of Zinc Deficiency in Children

Zinc deficiency is estimated at 17–20% of the global population [17,18,19] and it is estimated that 17.3% of preschool children in low- and middle-income countries are zinc deficient [20].

Zinc deficiency has significant clinical implications in the pediatric population, affecting multiple physiological systems. It impairs growth and development by disrupting cell proliferation and protein synthesis, primarily by reducing hepatic production of insulin-like growth factor-1 (IGF-1) [3]. Immunologically, zinc deficiency exacerbates immune dysfunction, increasing susceptibility to infections, including spontaneous bacterial peritonitis and other complications [11,21]. Zinc deficiency impairs both innate and adaptive immune responses, leading to cell-mediated immune dysfunctions that worsen outcomes in bacterial infections and sepsis [22]. Deficits in zinc adversely affect macrophage function, disrupting phagocytosis, intracellular killing, and cytokine production, which are critical for effective host defense [23]. Metabolically, zinc is crucial for ammonia detoxification via the urea cycle, and its deficiency worsens hyperammonemia and hepatic encephalopathy [7]. Moreover, zinc deficiency contributes to insulin resistance, hepatic steatosis, anorexia, and malabsorption, compounding nutritional deficits [24]. It also delays wound healing and compromises liver regeneration by impairing hepatocyte proliferation and the ZIP14 transporter pathway [25]. Deficiency compromises the liver’s ability to recover from injury, potentially accelerating the progression of liver disease.

Zinc deficiency is associated with a broad spectrum of clinical manifestations. Cutaneous and mucosal alterations are among the earliest and most characteristic features, including impaired wound healing, alopecia, acral dermatitis, stomatitis, cheilitis, and nail abnormalities, highlighting the importance of zinc for epithelial integrity and tissue repair. Immune dysfunction represents another major consequence, with impaired cell-mediated immunity predisposing affected individuals to recurrent infections. Gastrointestinal symptoms such as diarrhea, anorexia, and hypogeusia further exacerbate nutritional compromise and may contribute to a self-perpetuating cycle of malabsorption and deficiency. Endocrine- and growth-related disturbances, including growth retardation, delayed puberty, and hypogonadism, underscore zinc’s role in hormonal signaling and somatic development. Moreover, zinc deficiency adversely affects central nervous system function, manifesting as neurodevelopmental delay, behavioral disturbances, impaired concentration, and neurosensory deficits. Musculoskeletal involvement, characterized by reduced lean body mass and increased fracture risk, reflects altered bone metabolism, while during pregnancy, zinc deficiency has been linked to adverse fetal outcomes, including intrauterine growth restriction, low birth weight, and preterm delivery [26].

### 2.4. Zinc Deficiency in Cholestasis

Children with cholestatic liver disease had significantly lower serum zinc levels than healthy children [3,27]. Contributing factors include inadequate intake, malabsorption, and cytokine-mediated inflammation, leading to impaired growth, immune dysfunction, and increased health risks.

Zinc levels were positively associated with liver disease severity in children with chronic liver disease, including those with cholestatic disorders [28]. Also, children with cholestasis often require parenteral nutrition, which may be zinc-deficient, further exacerbating the condition. Cases of dermatitis associated with zinc deficiency have been documented in infants with cholestasis receiving parenteral nutrition lacking adequate zinc [29].

Zinc deficiency leads to multiple metabolic dysfunctions, including insulin resistance, hepatic steatosis, and hepatic encephalopathy in patients with chronic liver diseases, and also leads to inflammation and increased iron storage in the liver [2].

The mechanism of action of zinc primarily involves its binding to proteins, such as albumin and alpha-2-macroglobulin, as well as certain acids. This binding is directly proportional to zinc absorption efficiency. In progressive liver disease, declining albumin levels can significantly impair zinc transport and availability, thereby reducing zinc absorption and systemic bioavailability. This decline in zinc status may exacerbate hepatic dysfunction and related complications [30].

## 3. Selenium

Selenium is an essential trace mineral that plays a vital role in various physiological processes in the human body. This element is particularly important for children, as it supports proper growth, development, and immune function [31].

Although selenium deficiency is not common in developed countries, it can still be observed in certain areas with low selenium soil [32]. The prevalence of selenium deficiency is estimated at 28% [33] and varies across populations, clinical settings, and geographic regions, being a health concern in some areas of China and Africa, especially in sub-Saharan African countries (Ethiopia and Malawi). Also, selenium deficiency has been observed in Hungary, Switzerland, Poland, and some areas in Russia [31]. Selenium deficiency prevalence ranges from approximately 10% in general urban pediatric populations [34] to over 80% in high-risk clinical groups, particularly infants on parenteral nutrition [35].

Selenium serves numerous essential functions in the human body, acting as a cofactor for a variety of enzymes, known as selenoproteins, which are involved in antioxidant defense, thyroid hormone metabolism, and immune function, among other processes [31].

Although selenium is essential for human health, the margins between nutritional and toxic doses are very narrow, and excess intake can lead to adverse effects [36]. The human body cannot store selenium for extended periods, so maintaining adequate dietary intake is necessary to ensure proper physiological function [31]. However, daily intake recommendations for selenium vary between countries and organizations, making it difficult to establish universal guidelines [37].

Main dietary sources of selenium include meat, fish, grains, dairy, and certain vegetables. The selenium content of these foods is highly dependent on soil selenium, which is unevenly distributed [31]. Selenium is mainly absorbed in the duodenum and caecum through active transport, and its metabolism occurs in the liver, where hepatocytes play a crucial role [38].

In pediatric cholestasis, understanding the metabolism and clinical implications of selenium deficiency is important for disease management.

### 3.1. Functions of Selenium

Selenium is a key component of several essential selenoproteins. More than 30 types of selenoproteins have been identified, each with functions in various physiological processes [28]. These selenoproteins are involved in antioxidant defense, thyroid hormone metabolism, immune function, and DNA repair [28,39]. Some of the primary functions of selenium include:

- Antioxidant activity: Approximately half of selenoproteins exhibit antioxidant capabilities, protecting cells from oxidative damage and maintaining cell membrane integrity [37,40]. These selenoproteins include glutathione peroxidases, thioredoxin reductases, iodothyronine deionidases, selenoprotein P, Selenoprotein M, Selenoprotein H, Selenoprotein O, and Selenoprotein V [41]. These proteins employ selenocysteine, an amino acid, to catalyze reactions that neutralize reactive oxygen species and maintain cellular health. Glutathione peroxidases (GPXs) are among the most studied selenoproteins. GPXs are widely distributed throughout the body, and their activity levels reflect selenium status. Six types of GPXs are found in different cells and tissues; all, except GPX5, possess antioxidant activity [41]. Thioredoxin reductases, selenoproteins involved in the reduction of oxidized thioredoxin, regulate cellular redox state and protect against oxidative stress [41,42]. Iodothyronine deiodinases influence thyroid hormone metabolism and neutralize reactive oxygen species (ROS), maintaining redox balance and protecting thyroid epithelial cells [41]. Selenoprotein P functions as a selenium transporter, delivering selenium to various tissues and protecting against oxidative damage in the extracellular environment [43]. Selenoproteins H, M, O, T, and V also contribute to redox regulation and antioxidant defense, though their specific roles remain under investigation [41].
- Thyroid hormone metabolism. Selenium is essential for the function of deiodinase enzymes, which convert thyroid hormones like T4 into the more active T3 form. T3 interacts with cells to regulate metabolism, growth, and development. The synthesis and degradation of T3 depend on selenium-dependent iodothyronine deiodinases, of which there are three types: *DIO1*, *DIO2*, and *DIO3* [44]. These enzymes modulate thyroid hormone signaling by catalyzing the activation and inactivation of thyroid hormones, ensuring tissue-specific responses and maintaining homeostasis in circulating and intracellular thyroid hormone levels under varying physiological conditions [45]. The expression and activity of these deiodinase enzymes are influenced by selenium status, as adequate selenium is necessary for their proper functioning as a cofactor [45]. Other selenoproteins, such as glutathione peroxidases and thioredoxin reductases, indirectly support thyroid function by protecting the thyroid gland from oxidative damage.
- Immune function. Selenium plays a crucial role in enhancing both innate and adaptive immunity [46]. It improves the activity of various immune cells, such as macrophages and natural killer cells, which are essential for recognizing and eliminating invading pathogens. Selenium also significantly contributes to the adaptive immune response, which involves the activation of T and B lymphocytes [46]. This enhancement leads to a more effective, targeted response to specific pathogens, thereby improving the body’s ability to remember and respond to previously encountered infections.
- Additionally, selenium may contribute to other physiological processes, including DNA synthesis, reproduction, and cognitive function, although the evidence for these roles is less extensively explored [41].

### 3.2. Metabolism of Selenium

Selenium is primarily absorbed in the gastrointestinal tract, both in organic forms such as selenomethionine (SeMET) and selenocysteine (SeC) and in inorganic forms, including selenites and selenates. The metabolic processing of selenium is influenced by its chemical form; the organic form of selenium has demonstrated superior bioavailability for selenoprotein biosynthesis [47].

Its absorption efficiency under normal physiological conditions is between 70% and 90% [48]. Selenium is then transported in the bloodstream, bound to plasma proteins such as selenoprotein P and albumin. Selenium is then distributed to various tissues and organs, where it is incorporated into selenoproteins. The liver plays a central role in selenium metabolism, as it is involved in selenoprotein synthesis, selenium storage, and the regulation of selenium distribution to other tissues [49].

**Figure 2:**
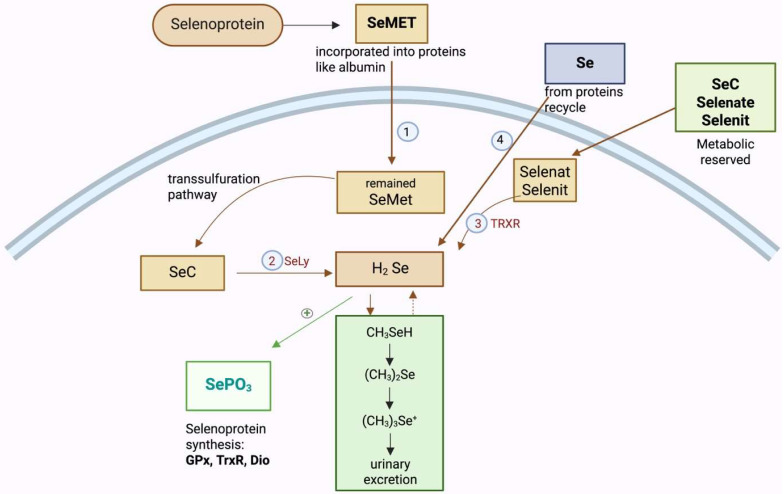
Metabolism of selenium in hepatocytes. The liver captures selenium from circulating sources, including dietary selenomethionine, selenium released through protein turnover, and inorganic forms such as selenite and selenate. Selenomethionine can be converted to selenocysteine via the transsulfuration pathway. Organic selenium (Sec) is degraded to hydrogen selenide (H_2_Se) by selenocysteine lyase (SCLY), while inorganic selenium is reduced to H_2_Se by thioredoxin reductase (TrxR). H_2_Se is then oxidized to selenophosphate (SePO_3_), the key precursor for selenocysteine synthesis in selenoproteins, including GPx, TrxR, and DIO. Excess H_2_Se is detoxified through methylation into volatile and urinary selenium metabolites. (Se—Selenium; SeMET—selemethionine; Sec—Selenocysteine; TrxR—Thiredoxin reductase pathway; H_2_Se—Hydrogen Selenide; SePO_3_—Selenophosphate) [48,49,50]. Created in BioRender. Adam, S. (2026) https://BioRender.com/aii76h1.

In the liver, all ingested selenium (organic or inorganic) is converted into hydrogen selenide (H_2_Se), the bioactive form. H_2_Se is transformed in the liver into selenophosphate (SePO_3_), which is crucial for the incorporation of selenium into selenoproteins as selenocysteine (SeC). Transport of selenium from the liver to other tissues for selenoprotein synthesis is mediated by selenoprotein P [50]. The liver converts excess selenium into dimethylselenide ((CH_3_)_2_Se)^−^, which is then excreted in the urine or via the breath [48]. A schematic representation of selenium metabolism in the liver is shown in Figure 2.

### 3.3. Clinical Implications of Selenium Deficiency in Children

Selenium deficiency can lead to various complications, such as weakened antioxidant defenses, compromised immune function, and impaired cell proliferation, all of which can contribute to the progression and severity of cholestatic liver disease [51]. In children, selenium deficiency can lead to hypothyroidism and impaired activity of thyroid hormones, which can affect growth, metabolism, and energy levels [52]. In growth and development, selenium deficiency has been linked to stunting, underweight status, and impaired cognitive performance in preschool children from rural Ethiopia [53]. Regarding immune function, selenium plays a critical role in defense against infection. Selenium supplementation may be an effective adjunct in the treatment of acute lower respiratory tract infections in children under 5 years, as selenium deficiency increases the pathology and severity of viral infections [54]. Adequate selenium status appears essential for optimal immune responses and endothelial function during acute systemic inflammation [55]. Also, selenium deficiency can lead to cardiovascular issues [28,56]. One of these entities is Keshan disease, which is characterized by a potentially fatal form of cardiomyopathy endemic to selenium-deficient regions of China [38,56].

In severely malnourish children, low selenium status and depressed glutathione peroxidase activity have been associated with hepatic dysfunction and increased cardiac failure risk [57]. Neurodevelopmental implications are recognized, with selenium deficiency linked to impaired neuronal development and poor cognitive performance [53,58]. Reduced concentrations of SELENOP were associated with intellectual disability [56,58,59].

In infants on long-term parenteral nutrition, selenium deficiency can lead to growth retardation, alopecia, and pseudoalbinism, which are reversible with appropriate selenium supplementation [31,32].

### 3.4. Selenium Deficiency in Children with Cholestasis

Cholestatic liver disease, such as biliary atresia, can impair selenium absorption and metabolism leading to decreased levels in the body [60]. In neonatal cholestasis, selenium deficiency has been reported in 13–33% of children [61]. Studies on selenium status in children with cholestasis specifically are limited. However, research on selenium status in patients with chronic liver disease, including cholestasis in adults, suggests that selenium levels are often lower in these patients compared to healthy individuals [28]. Dominik et al., in a study on 309 patients, adults with advanced chronic liver disease, found that 63% of patients were deficient in selenium, and the lower levels of selenium were correlated with disease severity [62]. Understanding the clinical implications of selenium deficiency in children requires further research to assess selenium levels in children with cholestasis to develop appropriate nutritional interventions and monitoring strategies.

## 4. Copper

Copper is an essential trace mineral for the body, acting as a cofactor for many enzymes, including cytochrome oxidase, superoxide dismutase, lysyl oxidase, ascorbic oxidase, dopamine-β-hydroxylase, ceruloplasmin, and tyrosinase [63]. The human body contains 1.4–2.1 mg/kg of copper. In children, the estimated average daily copper requirement is 0.26–0.68 mg, depending on age, and in adults, it is 0.70 mg/day [63]. The primary sources of copper are organ meats, shellfish, nuts, some grains, and water. Around 55–75% of the copper ingested daily is absorbed in the stomach and duodenum and transported to the liver via the portal vein. Intestinal absorption ranges from 12% to 71% in adults and 75% to 84% in infants [64]. A significant portion of the absorbed copper will bind to albumin and amino acids for transport to the liver via the portal circulation. Copper is absorbed into hepatocytes via the high-affinity copper transporter CTR1 located at the basolateral membrane. In the second phase, copper is transported to extrahepatic tissues by ceruloplasmin, albumin, or amino acids [65]. Together with ceruloplasmin, it forms a complex that is released into the systemic circulation and later distributed to the tissues (brain, kidneys, muscles, and connective tissues) [65].

### 4.1. Copper Metabolism

Copper metabolism is a tightly regulated, multifaceted biological process that encompasses uptake, distribution, sequestration, and excretion at both the cellular and systemic levels.

Mammalian enterocytes absorb dietary copper through a high-affinity, CTR1-mediated transport mechanism, after Cu^2+^ is reduced to monovalent cuprous ion (Cu^+^) in the presence of STEAP proteins [66]. Once internalized, cuprous (Cu^+^) ions are bound by copper chaperons such as CCS, COX17, and ATOX1, which direct them to specific cellular destinations. COX17 transports and delivers copper ions into the mitochondria; CCS (copper chaperone for SOD, superoxide dismutase) delivers copper ions to SOD1, which has an antioxidant role. ATOX1 (antioxidant protein 1) acquires Cu^+^ from CTR1 and then delivers it to the copper-transporting ATPases *ATP7A* and *ATP7B* (copper-exporting pumps) located in the trans Golgi network. When copper levels are low, ATPases retain copper in the Golgi apparatus; when copper levels are high, it is transported to the membrane for excretion by secretory vesicles or lysosomes. The function of *ATP7B* is to incorporate copper into ceruloplasmin and to export copper out of cells, into the biliary system, where it is subsequently eliminated in the stool [67].

**Figure 3:**
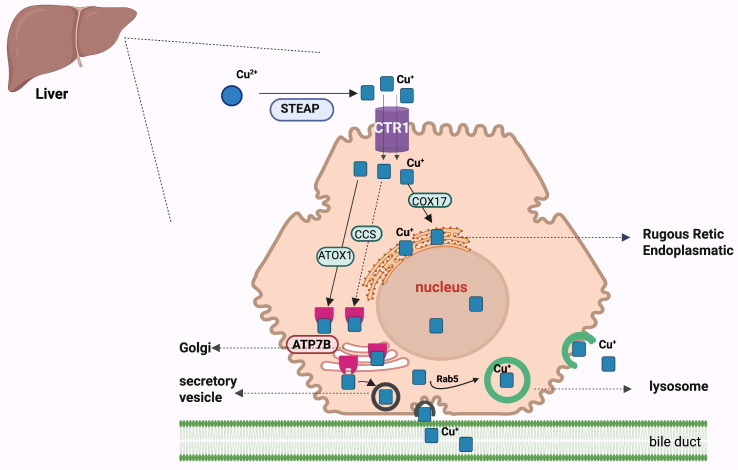
Metabolism of copper into hepatocytes. Dietary Cu^2+^ is reduced to Cu^+^ by STEAP metalloreductases, enabling high-affinity transport through the copper importer CTR1. Once inside the cytoplasm, Cu^+^ is distributed to specific intracellular pathways by dedicated copper chaperones. ATOX1 delivers Cu^+^ to the trans-Golgi network (TGN), where *ATP7B* (and *ATP7A* in non-hepatic cells) incorporates copper into cuproenzymes. COX17 transfers Cu^+^ to the mitochondrial intermembrane space for cytochrome c oxidase assembly, while CCS delivers Cu^+^ to cytosolic superoxide dismutase (SOD1). Under basal copper conditions, *ATP7B* remains in the TGN to support cuproenzyme maturation. When intracellular copper levels rise, *ATP7B* traffics to endolysosomal and apical vesicles—regulated in part by Rab5—to facilitate copper sequestration and biliary excretion. Together, these coordinated pathways maintain hepatic copper homeostasis and prevent copper-induced toxicity [66,67]. Created in BioRender. Adam, S. (2026) https://BioRender.com/kqzmrd3.

In peripheral tissues, copper is stored by MTs or directed to enzymes by chaperones such as ATOX1, COX17, and CCS, ensuring proper cellular function and avoiding toxicity [66]. Copper metabolism in hepatocytes is shown in Figure 3.

### 4.2. Functions of Copper

Copper is a trace element with a primary role in the proper functioning of enzymes involved in aerobic metabolism, such as mitochondrial cytochrome c enzymes, lysyl oxidase in connective tissue, dopamine β-monooxygenase in the brain, and ceruloplasmin. It also prevents the degradation of proteins, membrane lipids, and cellular nucleic acids, acting as a cofactor for apo-copper-zinc superoxide dismutase (apoCuZnSOD) and controlling the action of free oxygen radicals [68]. Copper participates in iron metabolism through proteins like ceruloplasmin [69].

The human body tightly regulates copper homeostasis to prevent both deficiency and toxicity. Inadequate copper levels can lead to a spectrum of clinical manifestations, including anemia, neutropenia, bone abnormalities, neurological deficits, and impaired immune function. At the same time, excessive accumulation can result in liver damage, neurological disorders, and renal impairment.

### 4.3. Copper in Cholestatic Diseases

Copper metabolism is significantly affected in cholestatic disorders, particularly in infants and children. In a study of intrahepatic cholestasis, both plasma and liver copper concentrations were higher than in patients with Wilson’s disease [70]. In the past, nutritional recommendations for children with cholestasis completely excluded copper from parenteral nutrition. Currently, it is recommended for use in parenteral nutrition solutions as it has been shown not to increase serum or hepatic copper levels [63].

The accumulation of Cu in the liver is considered a trademark of chronic cholestasis [71]. Infants with cholestatic diseases exhibit significantly higher hepatic and serum copper levels, associated with higher ceruloplasmin levels, compared with healthy control groups [72]. As cholestatic liver diseases progress, there is a gradual accumulation of the lighter 63Cu isotope in various organs, the serum, and the bones, resulting in copper dysmetabolism [73]. Excess copper in cells causes toxicity, as its redox properties can lead to marked formation of reactive oxygen species (ROS) that damage lipids, proteins, and nucleic acids [74,75]. This dysmetabolism associated with oxidative stress has been described in several neurodegenerative disorders [76]. Serum copper levels correlate with hepatic copper concentrations, making serum levels useful indicators of copper accumulation in hepatic cells [71,72]. The accumulation of copper and copper-binding proteins in periportal hepatocytes is an early indicator of chronic cholestasis. Histochemical analysis revealed this distinct pattern of copper distribution in various liver diseases [77]. Cholestasis does not appear to impair copper excretion enough to result in elevated levels; thus, cholestatic infants on parenteral nutrition may require copper doses higher than the standard dose [78]. Periodic monitoring of copper levels is recommended [71].

### 4.4. Clinical Implications of Copper Overload

The main disease involving disturbance of copper metabolism is Wilson disease, characterized by a defect in the ATP7B gene, leading to systemic copper overload and inefficient incorporation of copper into apoceruloplasmin, with a lack of biliary copper excretion [79]. In addition to liver accumulation of copper, deposits can also be found in the brain and eyes [80]. Copper overload in children with Wilson disease can lead to a spectrum of liver injuries, ranging from abnormal liver biochemical test results to cirrhosis or acute liver failure, typically manifesting within the first two decades of life [81,82]. Early detection and management of copper accumulation are therefore critical to minimize hepatic damage and improve patient outcomes [83].

In Wilson disease, chelation therapy with agents such as D-penicillamine and trientine is the main treatment and is most effective when started early [84,85]. However, in advanced stages, the prognosis remains guarded, even with aggressive chelation, underscoring the need for timely intervention [84]. In addition to chelation, zinc salts represent an alternative therapeutic approach. However, their efficacy in symptomatic patients with advanced liver disease remains controversial [86]. Liver transplantation is a viable option for children with end-stage liver disease secondary to copper overload, particularly when chelation therapy fails or is poorly tolerated or in acute liver failure due to Wilson disease [87].

Intrahepatic cholestasis of childhood, including patients with alpha-1 antitrypsin deficiency and liver disease, as well as patients with progressive familial intrahepatic cholestasis, is associated with impaired copper metabolism, resulting in markedly elevated hepatic copper levels with severe liver damage [70,88]. Because of bile duct obstruction, many patients with biliary atresia have impaired copper and zinc metabolism, and an excessive level of Cu is cytotoxic and results in liver fibrosis [82].

Patients with primary sclerosing cholangitis often present with abnormal copper metabolism. The majority have elevated hepatic copper levels and increased urinary copper excretion. Copper accumulation correlates with disease progression, with higher levels observed in advanced stages [89]. Primary biliary cholangitis is also associated with elevated hepatic copper levels, but its role in disease pathogenesis remains uncertain [30]. Studies have shown no correlation between liver copper levels and liver cell damage, suggesting copper may not be hepatotoxic in this particular disease [90].

Niemann-Pick C1 is an autosomal recessive lipid storage disorder caused by defects in the NPC1 gene. Recent studies have revealed a connection between copper metabolism and the NPC1 protein, which plays a vital role in intracellular copper transport and the incorporation of copper into ceruloplasmin [91,92]. Dietary copper restriction and copper chelation therapy have been shown to alleviate hepatic symptoms in NPC mouse models [93].

## 5. Manganese

Manganese is among the most common trace metals in the human body [6] which is mainly obtained from food and water. Dietary sources of manganese are diverse, with nuts, seeds, whole grains, legumes, and leafy green vegetables among the richest. The bioavailability of manganese, however, can be affected by various dietary factors, including phytates, calcium, and iron, which can inhibit its absorption in the gastrointestinal tract. Dietary manganese is absorbed at less than 5% through the gastrointestinal tract, then transported in the blood, bound to transferrin and albumin [6]. The highest levels of manganese in the human body are found in the liver, pancreas, bone, and brain [94].

Manganese exists in the human body in two biologically relevant oxidation states: Mn^2+^, the predominant form, and Mn^3+^. Among these, Mn^3+^ is a highly reactive oxidizing species; it can bind to transferrin or is usually reduced to the more stable form, Mn^2+^ [94].

While essential for life, manganese homeostasis is tightly regulated to prevent deficiency or excess, both of which can lead to adverse health outcomes. Manganese deficiency is rare, only in experimental conditions [6].

### 5.1. Functions of Manganese

Manganese is an essential trace element that functions predominantly as a cofactor and activator for a wide range of metalloenzymes, including arginase, glutamine synthetase, pyruvate carboxylase, and mitochondrial manganese superoxide dismutase (Mn-SOD). Mn-SOD plays a pivotal role in mitigating mitochondrial oxidative stress by catalyzing the dismutation of superoxide radicals into less reactive species [94]. Beyond its antioxidant function, manganese is involved in carbohydrate and lipid metabolism, enhances protein synthesis, and supports the biosynthesis of vitamins C and B [95]. It also participates in enzymatic pathways critical for hematopoiesis, regulates endocrine activity, contributes to bone and connective tissue formation, and is essential for skeletal development, reproductive function, and immune modulation [95].

### 5.2. Metabolism of Manganese

Manganese absorption primarily occurs in the small intestine, involving both active and passive transport mechanisms [96,97]. Once absorbed, it enters the bloodstream and is quickly distributed to various tissues.

Hepatocytes play a central role in systemic manganese homeostasis by coordinating its uptake, intracellular handling, and biliary excretion. Although DMT1 (divalent metal transporter-1) is essential for intestinal manganese absorption, its contribution to hepatic uptake is minimal. Instead, hepatocytes primarily rely on two complementary pathways: the transferrin–transferrin receptor (Tf–TfR) system and the ZIP family transporters [94,97].

Circulating trivalent manganese (Mn^3+^) binds to transferrin synthesized and secreted by the liver. The Mn^3+^–Tf complex is internalized by hepatocytes via TfR-mediated endocytosis. Within endosomes, Mn^3+^ is released and reduced to its bioactive divalent form (Mn^2+^), enabling its entry into the cytosol for metabolic utilization [97].

The predominant route for hepatic manganese uptake is mediated by the metal transporters ZIP14 (*SLC39A14*) and ZIP8 (*SLC39A8*), located on the basolateral and apical membranes, respectively [98]. ZIP14 is crucial for systemic manganese clearance: it removes excess manganese from the bloodstream, thereby preventing toxic accumulation in extrahepatic tissues, particularly the brain [99]. Conversely, ZIP8 contributes to manganese conservation by reclaiming it from bile, a mechanism that becomes especially important under conditions of low dietary manganese intake [99].

**Figure 4:**
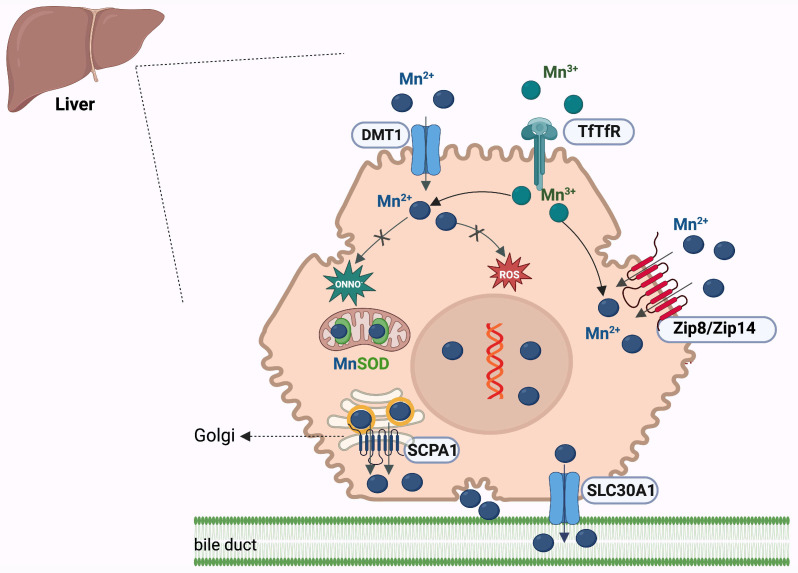
Metabolism of manganese in hepatocytes. Manganese enters hepatocytes primarily as Mn^2+^ via DMT1 and as Mn^3+^ through transferrin receptor–mediated uptake. Mn^2+^ is distributed to cytosolic and mitochondrial compartments, where it supports MnSOD activity and other manganese-dependent enzymes, while excess Manganese promotes reactive oxygen species formation. ZIP8/ZIP14 facilitate additional Mn^2+^ influx. Intracellular manganese is trafficked via vesicular pathways and directed toward biliary excretion via transporters, including *SLC30A1* and *SCPA1*, thereby maintaining systemic manganese homeostasis [96,97,99,100]. Created in BioRender. Adam, S. (2026) https://BioRender.com/p5p26ab.

Manganese efflux from hepatocytes is regulated primarily by ZnT10, a transporter that functions in concert with ZIP14 to export excess manganese into bile [98,100]. This coordinated ZIP14–ZnT10 axis is essential for maintaining manganese homeostasis, and genetic disruption of either transporter results in severe manganese overload syndromes [101]. Manganese metabolism in hepatocytes is shown in Figure 4.

### 5.3. Clinical Implications of Manganese in Cholestasis

In children with cholestasis, the compromised biliary excretion pathway results in elevated serum manganese levels [60]. Manganese can then cross the blood–brain barrier and accumulate in the brain, particularly in the basal ganglia.

Manganese accumulation has neurotoxic effects manifested with Parkinsonian symptoms and cognitive deficits [102,103,104,105]. Manganese-induced neurotoxicity involves dysregulation of mitochondrial dynamics, oxidative stress, and excitotoxicity, leading to neuroinflammation and neuronal death [105]. In children, studies have shown that manganese exposure is associated with cognitive, motor, and behavioral deficits [106].

Also, elevated manganese has been associated with hepatocellular injury, bile duct and portal fibrosis, suggesting a potential pathogenic role in liver damage [60]. High manganese intake in neonates receiving parental nutrition is likely a significant factor contributing to the development of cholestasis [107].

In biliary atresia, the risk of subclinical manganese neurotoxicity highlights the importance of monitoring, with serial serum manganese levels and brain MRI proposed for early detection. Manganese may be a promising biomarker for assessing liver function in pediatric patients with BA [60].

## 6. Management Strategies in Cholestasis

Effective management of the trace elements in patients with cholestasis requires a personalized, multidisciplinary approach that incorporates tailored nutritional interventions, careful monitoring, and evidence-based adjustments to both parenteral and enteral nutrition regimens. Coordination between hepatologists, dietitians, neurologists, and transplant teams ensures holistic management of hepatic, nutritional, and neurodevelopmental complications.

In patients requiring parenteral nutrition, trace element supplementation must be carefully individualized to avoid toxicity while addressing deficiencies. There is no consensus on pediatric dosing. Restriction of copper is not routinely recommended, as this is a controversial issue in cholestatic patients [108]. In preterm infants with high copper requirements, ESPGHAN and ESPEN guidelines recommend a double dose (from 20 to 40 μg/kg/day) [109]. Due to the risk of neurotoxicity, manganese should be limited to 1 µg/kg/day, with a maximum of 55 µg/day [109,110]. Standard supplementation recommendations include 2–3 μg/kg/day of selenium, with a maximum of 60–100 μg/day, and 50–500 μg/kg/day according to age, and a maximum dose of 5–6.5 mg/day of zinc [109,110].

For patients receiving enteral nutrition, oral trace element supplementation is essential to address subclinical or overt deficiencies. Supplementation with 1–2 mg/kg/day of zinc sulfate is recommended, particularly in the presence of growth retardation, diarrhea, or acrodermatitis; further validation of the reference is warranted [61,111]. Recommended selenium doses are adjusted according to age and the severity of malabsorption or gastrointestinal losses. Normally, doses are lower than those used in parenteral nutrition and are based on adequate intake (AI) recommendations, approximately 1–2 µg/kg/day. In cases of severe cholestasis, with significant enteric losses or increased oxidative stress, the dose may be increased to 2–3 µg/kg/day. Still, serum monitoring is essential to avoid deficiency or overdosing [31,112,113].

Ongoing surveillance of trace element status and nutritional impact is critical. Every 3–6 months, serum copper, zinc, manganese, selenium, ceruloplasmin, 25-hydroxyvitamin D, and vitamin E are measured [60]. Annually, bone health should be assessed via dual-energy X-ray absorptiometry (DEXA), and a brain MRI should be considered in patients with elevated serum Mn to evaluate potential neurotoxicity [114]. In Table 1, we present the main effects of trace element disturbances, recommended nutritional doses, and parameters to be monitored.

Although zinc and selenium deficiency appear to be a shared feature across different etiologies of pediatric cholestasis, disturbances in copper and manganese homeostasis show greater variability and are more closely related to the degree and chronicity of cholestatic bile flow impairment. In obstructive cholestasis, such as biliary atresia, early and sustained disruption of bile acid–dependent excretory pathways promotes hepatic copper accumulation and manganese retention, reflecting impaired biliary clearance of these elements [60]. In contrast, intrahepatic genetic cholestasis (e.g., PFIC) is characterized by primary defects in bile acid transporters, with trace element disturbances evolving more gradually and becoming prominent mainly in advanced disease stages [119]. These observations suggest that trace element imbalance in pediatric cholestasis arises predominantly from altered bile acid–mediated absorption and excretion, hepatic retention, and inflammation-driven redistribution, rather than from etiology-specific regulation of trace element transporters per se [120].

To provide a structured overview of available data, reported pediatric reference intervals and concentrations described in cholestatic liver disease are summarized in Table 2. However, it is important to emphasize that no universally validated, disease-specific clinical thresholds currently exist for pediatric cholestasis, particularly for hepatic concentrations. Reported values should therefore be interpreted within the clinical context and methodological framework of each study.

## 7. Controversies, Emerging Therapies, and Future Directions

The management of trace elements in cholestasis, particularly in the context of parenteral nutrition, remains an evolving area of clinical and translational research. Historical restrictions on copper supplementation in parenteral nutrition are challenged by studies showing that copper deficiency poses risks [63]. Similarly, while manganese neurotoxicity remains a concern, the use of EDTA-based chelation remains experimental and lacks standardized clinical application [102]. Modulation of the gut microbiome with probiotics, such as Lactobacillus spp., has shown promise for enhancing bile acid metabolism and improving zinc absorption. This effect may be further augmented by fecal microbiota transplantation [123]. The development of non-invasive biomarkers, such as fecal manganese excretion and urinary zinc markers, could facilitate earlier detection of trace element imbalances and allow for individualized treatment strategies. Advances in gene therapy, including targeting *ATP7B*—the copper transporter mutated in progressive familial intrahepatic cholestasis—and exploring polymorphisms in genes encoding metal-binding proteins (e.g., *SLC30A10* for manganese), hold potential for genotype-guided interventions. Moreover, establishing international registries is critical for standardizing diagnostic and therapeutic protocols across centers [104]. Looking ahead, innovative approaches combining targeted chelation therapies for copper and manganese overload with gene-based correction of inherited defects, such as progressive familial intrahepatic cholestasis, may redefine treatment paradigms and represent a promising frontier in the field.

## 8. Conclusions

Cholestasis, resulting from impaired bile flow, disrupts liver function and overall balance, significantly affecting trace element metabolism. Additionally, inflammation from cholestasis can increase levels of inflammatory cytokines, such as tumor necrosis factor-alpha, interleukin 1-β, and IL-6, further disrupting trace element balance. The impact of cholestasis on trace element status extends beyond simple deficiencies, often leading to complex redistribution patterns across tissues and body fluids. These alterations can have far-reaching consequences for various physiological processes, including immune function, antioxidant defense, and neurological function.

The management of trace element imbalances in pediatric cholestasis epitomizes the intersection of precision medicine and nutritional science. Present therapeutic approaches are centered on mitigating deficiencies and preventing toxicity. Current research underscores the need for innovative diagnostic and therapeutic approaches. By integrating clinical monitoring, genetic insights, and innovative diagnostics and therapies, clinicians can improve quality of life and long-term outcomes for these vulnerable patients. Future directions should emphasize multicenter collaborative studies to standardize protocols and address gaps in our understanding of trace element dynamics in cholestatic liver disease.

## Figures and Tables

**Table 1:** Summarization of the main effects of trace elements deficiency/overload, as well as doses in parenteral or enteral nutrition, and monitoring parameters.

Elements	Deficiency/Overload	Doses	Monitoring
Zinc	Deficiency: Reduced protein synthesis Increased susceptibility to infections Insulin resistance and hepatic steatosis	Parenteral nutrition [115] <3 months: 250 µg/kg/day >3 months: 100 µg/kg/day. Older Children/: 50 µg/kg/day (max 5 mg/day). High-Output GI Losses: 200–500 µg/kg/day Enteral nutrition [116] Preterm: 1–3 mg/kg/day 0–6 months: 2.0 mg/day 7 months–3 years: 3.0 mg/day 4–8 years: 5.0 mg/day 9–13 years: 8.0 mg/day	Zn supplementation can reduce serum copper concentrations by inhibiting its absorption. This can potentially lead to copper deficiency, resulting in pancytopenia and neuropathy. Regular monitoring of Zn concentrations, alongside copper concentrations and complete blood count assessments, is advised to detect toxicity early in patients on long-term PN [117].
Selenium	Deficiency: Hypothyroidism Neurodevelopmental disease Cardiovascular disease	Parenteral [110] Preterm/Neonates: 2 µg/kg/day (1.5–4.5 µg/kg/day) Infants and children: 2 µg/kg/day Children and adolescents: 40–60 µg/day (max: 100 µg/day) Enteral: 1.3–3 µg/kg/day [110]	Periodic monitoring of selenium plasma levels and inflammatory markers in PN. Systemic inflammation decreases serum selenium levels. Renal function must also be monitored, due to renal excretion of selenium [118]
Copper	Overload: Chronic liver disease/end-stage liver disease Neurological impairment Deficiency: Hematologic dysfunction (neutropenia, anemia)	Parenteral nutrition [110] 20 µg/kg (max 50 µg/day) In children with cholestasis, the dose should be lowered.	Periodic monitoring of serum copper and ceruloplasmin. Risk of overload in patients with associated cholestasis.
Manganese	Overload: Neurological disease (Parkinsonian symptoms)	Parenteral nutrition [108] 1 µg/kg/day (max 55 µg/day) No supplementation in cholestasis	Periodic monitoring of serum levels, due to the risk of overload.

**Table 2:** Reported serum and hepatic concentrations of zinc, selenium, copper, and manganese in pediatric populations, including cholestatic liver disease.

Trace Element	Biological Matrix	Reported Concentrations *	Pediatric Population/Clinical Context
Zinc (Zn)	Serum	~64–124 µg/dL [121]	Pediatric reference intervals
	Serum	Reduced [60]	Children with biliary atresia
Selenium (Se)	Serum	~50–120 µg/L [37]	General pediatric reference range
	Serum	Reduced levels reported [60]	Infants with biliary atresia
Copper (Cu)	Serum	~57–153 µg/dL [121]	Pediatric reference intervals
	Serum	Normal or elevated [60,63,72]	Pediatric cholestatic liver disease
	Liver	Increased hepatic copper accumulation [70]	Pediatric chronic cholestasis
Manganese (Mn)	Whole blood	~4–14 µg/L [122]	Pediatric reference values
	Serum	Increased levels reported [60]	Pediatric cholestasis
	Liver/brain	Tissue accumulation associated with neurotoxicity [106]	Pediatric chronic cholestasis

Note: * Reported values represent pediatric reference intervals or ranges described in the literature. Currently, no universally validated pediatric disease-specific clinical thresholds for trace elements in cholestatic liver disease, particularly for hepatic concentrations, have been established. Available cut-offs are largely extrapolated from adult data or derived from population-based reference ranges and may vary according to age, inflammatory status, disease severity, nutritional intake, biological matrix (serum, plasma, whole blood, or tissue), and analytical methodology.

## Data Availability

No new data were created or analyzed in this study. Data sharing is not applicable to this article.

## Abbreviations

The following abbreviations are used in this manuscript:

(CH_3_)_2_Se	Dimethyl selenide
^63^Cu isotope	Stable copper isotope
apoCuZnSOD	Apo-copper-zinc superoxide dismutase
ATP7A	ATPase Copper-transporting Alpha
ATP7B	ATPase Copper-transporting Beta
ATPases	large family of enzymes
ATOX1	Antioxidant protein 1, a copper chaperone
BA	Biliary atresia
CCS	Copper chaperone for superoxide dismutase
COX17	cytochrome c oxidase copper chaperone 17
Cr	Chromium
CTR1	Copper transporter 1
Cu	Copper
Cu^+^	Monovalent copper
Cu^2+^	Divalent copper
DEXA	Dual-energy X-ray absorptiometry
DIO	Iodothyronine deiodinases
DIO1	Type 1 iodothyronine deiodinase
DIO2	Type 2 iodothyronine deiodinase
DIO3	Type 3 iodothyronine deiodinase
DMT1	Divalent metal transporter 1
DNA	Deoxyribonucleic acid
EDTA	Ethylenediaminetetraacetic acid, a metal chelating agent
ESPGHAN	European Society for Paediatric Gastroenterology, Hepatology and Nutrition
ESPEN	European Society for Clinical Nutrition and Metabolism
Fe	Iron
GPx/PXs	Glutathione peroxidase/glutathione peroxidase family
GPX5	Glutathione peroxidase 5
H_2_Se	Hydrogen selenide, a central intermediate in selenium metabolism
IGF-1	Insulin-like growth factor 1
IL-6	Interleukin-6
I	Iodine
Mn	Manganese
Mn^2+^	Divalent manganese
Mn^3+^	Trivalent manganese
Mn–SOD	Manganese-dependent superoxide dismutase (SOD2)
Mn^3+^–Tf complex	Manganese–transferrin complex
Mo	Molybdenum
MRI	Magnetic resonance imaging
mRNA	Messenger ribonucleic acid
MTF1	Metal-regulatory transcription factor 1
MTs	Metallothioneins
NF-κB	Nuclear factor kappa B
ONOO^−^	Nitric oxide
p53	Tumor suppressor protein p53
Rab5	Small GTPase involved in early endosomal trafficking
RNA	Ribonucleic acid
ROS	Reactive oxygen species
SCLY	Selenocysteine lyase
Se	Selenium
SeC (Sec)	Selenocysteine
SeMET	Selenomethionine
SePO_3_	Selenophosphate
SCPA1	Secretory Pathway Ca^2+^-transporting ATPase 1
SOD	Superoxide dismutase
SOD1	Copper/zinc superoxide dismutase
STEAP	Six-transmembrane epithelial antigen of the prostate, a metal reductase
T3	Triiodothyronine
T4	Thyroxine
Tf	Transferrin
TfR	Transferrin receptor
Tf–TfR	Transferrin–transferrin receptor complex
TGN	Trans-Golgi network
TrxR (TRXR)	Thioredoxin reductase
Zn	Zinc
ZnT (SLC30)	Zinc transporter family
ZnT10 (SLC30A10)	Zinc and manganese efflux transporter
ZIP (SLC39)	Zrt-/Irt-like protein family of metal influx transporters
ZIP4	Intestinal zinc transporter
ZIP8 (SLC39A8)	Zinc and manganese transporter
ZIP10	Zinc transporter involved in immune and cellular signaling
ZIP14 (SLC39A14)	Hepatic zinc and manganese transporter
ZIP family transporters	Family of zinc influx transporters
